# Water-constrained green development framework based on economically-allocable water resources

**DOI:** 10.1038/s41598-023-31550-7

**Published:** 2023-03-31

**Authors:** Eisa Bozorgzadeh, S. Jamshid Mousavi

**Affiliations:** 1 School of Civil and Environmental Engineering, Amirkabir University of Technology (Tehran Polytechnic), Tehran, Iran; 2 School of Civil and Environmental Engineering, Amirkabir University of Technology, Hafez St., Tehran, Iran

**Keywords:** Environmental economics, Socioeconomic scenarios, Sustainability

## Abstract

Water as a main driver for sustainable development (SD) should be optimally allocated to different users to support economic, social, and environmental functions. Traditional approaches are not able to account for all the mentioned functions simultaneously, therefore a change in the allocation approaches is necessary. This paper proposes a new framework for inter-sectoral water allocation called “water-constrained green development” (WCGD) to better meet the SD goals. The framework optimally allocates economically-allocable water (EAW), which is the total available water resources left after subtracting the amount of water required for drinking, sanitation, and environment (DSE), to different job classes. It was tested in Sistan Region- a low-developed area in southeast of Iran- which stands on agriculture. In the recent years, because of water crisis, intensity of dust problem, lack of sustained occupation, and immigration, the region’s rate of population growth has been negative. Also, due to decrease of Helmand River inflow, Hamoun wetland, being the major source of food and shelter for the Sistan’s residents, has been degraded. Therefore, Sistan Region needs to take a new development route. The shares of occupation and gross domestic product (GDP) in the agricultural sector of Sistan are respectively 29.1 and 14.8%, whereas they are on average 1 and 7% in Iran. Application of the proposed framework in Sistan Region under three scenarios of available EAW resources showed that the optimal reallocation of water among 15 job classes can improve job availability and GDP of the region currently suffering from poor economy and employment conditions. Based on the optimal job pattern obtained, the share of GDP of Sistan’s agricultural sector drops to 7.1% while the shares of industrial and service sectors increase respectively from 9.7 and 75.4% to 13.7 and 79.2%, which are close to those of the country averages. Also, under the WCGD-based optimal solution, 68, 14, and 18% of people will respectively be employed in service, industry, and agriculture sectors. Additionally, the total available jobs and GDP will increase by 8.9 and 51.1%, respectively, leading to improved socio-economic well-being of  the region’s people and protection of its environmental resources.

## Introduction

Water is an important driver of development and should be used for sustainable development^1^. The term "sustainable development" (SD) was first coined by the World Commission on Environmental Development (WCED) in the Brundtland Commission^2^. To achieve SD, a balance must be struck among the economic development, environmental responsibility and social development^3^.

“Green growth” as a technical approach to meeting SD goals means fostering economic growth while ensuring that natural assets continue to provide the required resources and environmental services on which our well-being relies^4^. According to green growth, the traditional attitude to the development must change to a more sustainable approach. Indeed, development planning is a complicated task because of various interconnected factors^5^ and many uncertainties regarding possible changes in the future^6^. In this complicated task, water and its related system are considered as important components. On the other hand, interconnectedness of water systems components is an important feature that must be considered in the process of water resources management^5^. The framework of water-constrained green development (WCGD) presented in the current study is based on the concept of green growth focusing on socio-economically exchange of water among different users. It reflects the important role of water in GDP of agriculture, industry, and urban services as well as environment protection and socio-political stability. WCGD benefits from the principles of spatial planning to design the development strategies and programs. Spatial planning helps the development process with the identification of natural assets. Adams et al.^7^ believe that regional spatial design is a key tool for the development of sustainable climate adaptation strategies. Meerow and Newell^8^ presented a case study of spatial design for green infrastructure in Detroit. They developed a green infrastructure spatial planning model to maximize ecosystem services and reveal exchanges, subscribers, and key points for the future of green infrastructure. Novak et al.^9^ studied the role of local spatial plans in the countries of Central and Eastern Europe and found that there are major analogies regarding the legal formula of local spatial plans and the practical problems involved. Indeed, spatial planning has an important role in integrating the use and management of land and water resources^10^ and in water supply^11^, so water is one major part of spatial planning. Goswami et al.^1^ developed a comprehensive model by adding spatial planning and economic analysis to the food-energy-water nexus framework. Gallagher et al.^12^ emphasized the special role of water in green growth and its relationship with economic activities, ecosystem functions, and social health. Hu et al.^13^ proposed a multi-objective model for equitable water allocation, economic efficiency, and risk control. In this study, the Gini coefficient was used to optimize equitable water allocation among agricultural, urban, and industrial sectors. Wang et al.^14^ proposed a model for optimal allocation of water resources based on water security. The goal was to minimize the length of water shortage period in long term. For this purpose, an indicator called "water resources security" was presented. Optimizing the cultivation pattern in the agricultural sector has been one of the most interesting field of study in development planning^15–17^. Osama et al.^17^ evaluated the cropping pattern of Egypt from 2008 to 2012 and used a linear programming (LP) model to investigate the optimal pattern of crops. According to the results, modification of the existing cropping pattern could increase the gross net return without adding any other expenses. Hao et al.^18^ considered the uncertainty of water availability and water saving potential aiming to maximize agricultural net benefit per unit of irrigation water in Heihe River Basin. They showed that if the irrigation water-saving increases by 10%, the net water-saving quantity will increase by 21.5–22.5 million cubic meters (mcm) and the gross water-saving quantity will increase by 275.7–303.0 mcm. Yazdandoost et al.^19^ used the virtual water concept to show the effectiveness its consideration in agricultural activities. Using a multi-objective optimization model, they provided a revised cropping pattern in the Urmia Lake Basin. To determine the optimum cropping pattern for Kermanshah Plain, Iran, Barati et al.^16^ used a multi-objective optimization model considering different scenarios. Results showed that in order to optimize the cropping pattern, the area allocated to wheat, barley, grain and forage maize, tomato, clover, and onion should be decreased and the area of saffron, rose, greenhouse, medicinal plants, and olive should be decreased. Jin and Ge^20^ used a linear programming model to optimally allocate the land resources using a water quality dynamic monitoring model, and showed that the water shortage rate reduced to 25%. Ajudiya et al.^21^ developed the teaching learning-based optimization (TLBO) approach for allocation of water in a multiple reservoir system to optimize cropping pattern and maximize the resulting net benefits. TLBO application increased the net benefit as much as 20.24% in comparison with an LP model.

In addition to the optimal use of water and land resources, some researchers also paid attention to human resources and social factors. Habibi Davijani et al.^22^ studied the issue of water resources allocation with the aim of maximizing employment in the agricultural and industrial sectors. In this research, labor is mentioned as the most important factor in production and employment. However, social acceptability that plays an important role in spatial planning is not considered in this work. Amini Fasakhodi et al.^23^ investigated the sustainability of water resources and the optimal cultivation pattern in agricultural systems by fractional goal programming in which two objective functions were considered: (1) net return to water consumption and (2) number of job opportunities created by water consumption. Viola et al.^24^ studied the challenges regarding green employment creation in agriculture, and evaluated the employment of labor in green industries, labor market, and the present state of the education system. They finally provided solutions to the required training problem within the process of green development. Cecere and Mazzanti^25^ studied green jobs and environmentally-sound innovations in small and medium-sized European companies. Because of neglecting the link between the economic sectors, they noted that the roon for development was small. To search for the best cropping pattern in Heihe River basin, China. He et al.^26^ proposed an optimization model based on a distributed water consumption model of  crop suitability. Results showed that although water consumption increased slightly in the proposed optimal plan, the income values per unit water and unit area increased significantly compared to current conditions.

Although development processes have been extensively evaluated by many researchers, most of studies have neglected the links among the main economic sectors of agriculture, industry, and service. The present study is an attempt to make a link among those basic sectors through the proposed WCGD framework covering a larger part of the development space and processes. In this framework, by considering water exchanges among the economic sectors, the interactions between limited water resources and the development indicators are evaluated considering the three pillars of SD (society, environment, and economy). In this regard, the concept of economically-allocable water (EAW) is introduced to distinguish the amount of water resoiurces that can economically be allocated from that required for drinking, sanitation, and environment (DSE), ensuring the sustainability of the environment and the society. Therefore, the WCGD framework explicitly accounts for socio-environmental factors in the development process. Additionally, to guarantee the possibility and acceptability of the resulted plan, the development programs in WCGD are extracted from spatial planning studies. In the green development pronciples are used in WCGD to achieve the maximum economic profit, the least destructive environmental effects, and social acceptability through better employment conditions.

## Study area

**Figure 1:**
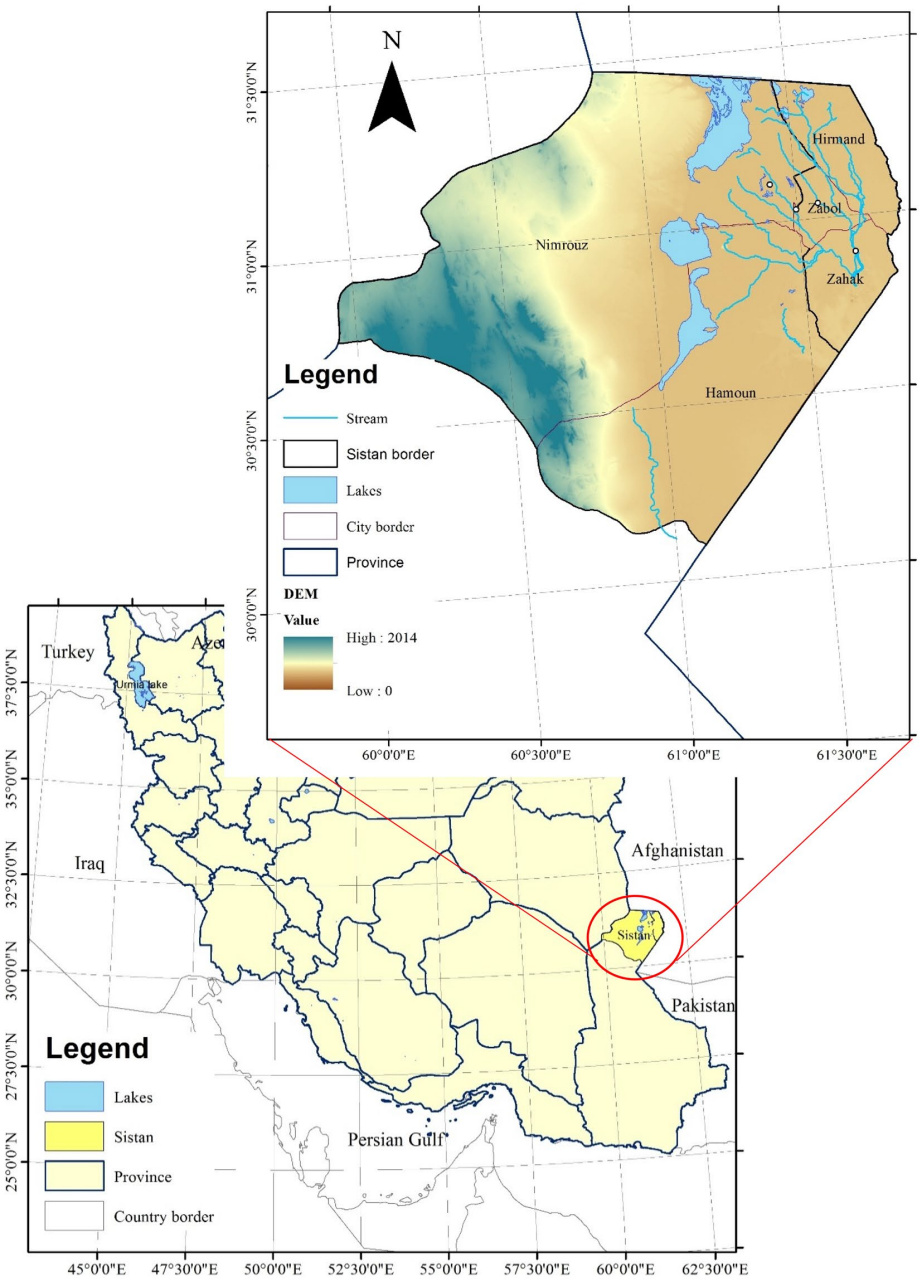
Location of Sistan Region in Iran (This figure was created in the Quantum GIS, version 3.12.2).

Sistan plain is part of the Helmand River catchment in southeastern Iran. Helmand River with an area of 367,000 km^2^ contains 47% of Afghanistan and 11% of its total water resources where about 10% of the Helmand Basin is located in Iran (Fig. 1). The average annual rainfall of the basin is 160 mm, while it is about 50 mm in the lower Sistan plain^27,28^. Sistan Region in the north of Sistan-Baluchestan province (SB) of Iran with a hot and dry climate is located between latitudes 30° 10′ and 31° 28′ North and longitudes 58° 40′ and 61° 45′ East. The 120-day winds and quick sands with a height of about 2 m are among the main features of Sistan Region^27,29^. About 200,000 ha of land in this plain has agricultural potential, and can be included in the crop production cycle^29^. Agriculture is the main economic activity of SB. It accounts for 29% of Sistan's employment, where only 17.6% of Iran's total employment is in the agricultural sector. Unemployment percentage in SB is 18.6, while it is on average 12.1 for the country^30^. The area of Sistan Region is 15,171 km^2^ consisting of 5 major cities: Zabol, Zahak, Nimrouz, Hamoun, and Hirmand (Fig. 1). Soukhteh city, an archaeological site of a sizable Bronze Age urban settlement, is the main tourist attraction of Sistan Region located near Hamoun city. The earliest known animation of the world was discovered in this site^31^. The city was placed on the UNESCO World Heritage List in June 2014. The climate of Sistan desert, which is hot and dry with wet period of cold season, is similar to that of Iran’s inland plateau. The average temperature is 22 °C and its relative humidity varies between 28 and 46% with an average of 38.2%^32^. Annual evaporation is about 4600 mm, 70% of which occurs when 120-day winds take place^29^. The main source of water in the region is the inflow of Helmand River from Afghanistan. Unfortunately, this source of water has fluctuated in the recent years resulting in a collapse of the agriculture as the main field of occupation^33^.

## Methodology

### WCGD framework

**Figure 2:**
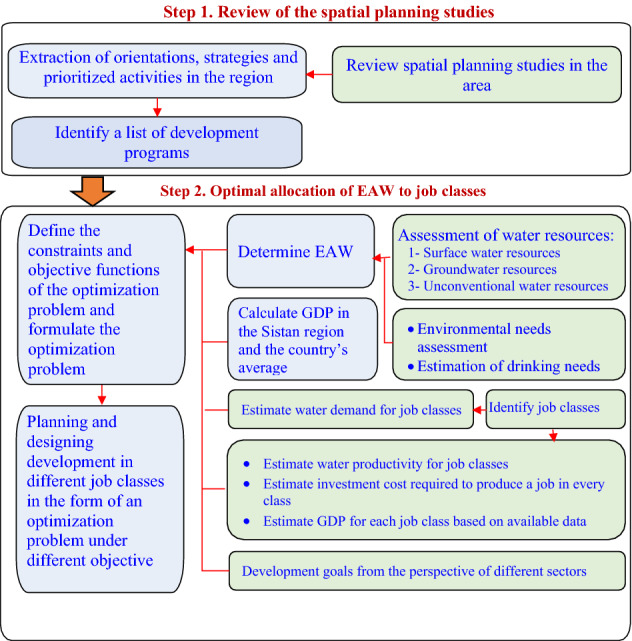
The proposed framework and research methodology.

The approach of the present article is based upon setting an optimal development plan paying attention to the natural assets and restrictions of the region. An optimization model is used to allocate the natural assets and resources of the region including water, soil, capital, and manpower to different job classes. To achieve the goal, several steps are defined according to the flowchart presented in Fig. 2.

As shown in Fig. 2, the proposed methodology consists of two main steps. In the first step, the available development plans are reviewed and the strategies and plans are extracted. This step provides a list of activities in different economic sectors to feed the optimization model as the decision space. In the second step, a comprehensive assessment of natural resources and economic productions is done, then by calculating the economically-allocating water (EAW) and defining an idle vision to the study area, a reallocation of water to the economic sectors is performed. Here, a simulation model is developed and the reallocation results are provided for several scenarios of development. Finally, the reallocated water in every sector is taken, several job classes are defined according to the list of activities extracted in the first step, and the optimal allocation of water to different job classes are calculated using a genetic algorithm optimization model. More details on some of the key steps explained above are provided in the following sections.

### Determining the development potentials and programs

Key inputs to the proposed model are a set of development programs presented typically in the spatial planning reports where a variety of programs are offered in different economic sectors of industry, agriculture, and service. According to the spatial planning of SB Province^34^, two important policies should be followed including population stabilization and the effective relationship with neighboring countries, so the main proposed strategy is to focus on water economy and transit activities. In the mentioned spatial planning documents, development in industrial and agricultural sectors has been emphasized according to the province potentials and interactions with other parts of the country. Meanwhile, the following practical plans have been highlighted in the spatial planning studies of Sistan Region^34^:

1. Improving agricultural cropping pattern by cultivating more profitable crops such as grapes, oilseeds, melons, citrus, and medicinal plants.
2. Improving irrigation efficiency in orchards (500 ha per year).
3. Increasing water transfer efficiency by improving and completing irrigation canals.
4. Fisheries and investment on ornamental fish in Zabol and Zahak (Fig. 1).
5. Development of agricultural products and food, metal, chemical, non-metal mineral, cellulose, and textile industries in Zabol and Zahak.
6. Mining of chromite metal and non-metal minerals of decorative building stones in Zabol.
7. Enhancing the capacity for clean energy generation, especially wind and solar energy.
8. Construction of a tourist village in Hamoun (Fig. 1) and development of historical tourism and ecotourism, desert tourism, off-road hydrotherapy, mounting, aviation and navigation, and paying attention to the historical area of Soukhteh City.
9. Desalination for drinking and industrial water supply in Zabol.

Obviously, any future plan for the Region should be in line with the above strategies. Hence, cropping pattern change from cultivating crops with high water demands and low economic values to the ones having lower water demands and higher economic values and improving irrigation efficiency in the agricultural sector should be considered. On the other hand, investment on the industrial activities mentioned in above strategies should also be considered. Moreover, several programs in the service sector such as tourism and recreational activities are important to be established in Sistan Region. Therefore, indicators reflecting these programs should be involved in the proposed WCGD-based optimization model developed in this study.

### Analysis of water resources and water demands

The proposed WCGD framework is based on the EAW concept accounting for socio-environmental aspect of SD that recognizes water-related basic, vital rights of humans and the nature to be met. The remaining water is considered as an economic commodity to be used economically efficient. EAW is therefore equal to the total available water resources, including surface water, groundwater, and unconventional water, subtracted by potable and environmental water needs. Note that the amount of potable water required is calculated based on the per capita water consumption and the target population at the end of planning horizon according to the spatial planning studies of the region.

#### Water resources of Sistan

**Figure 3:**
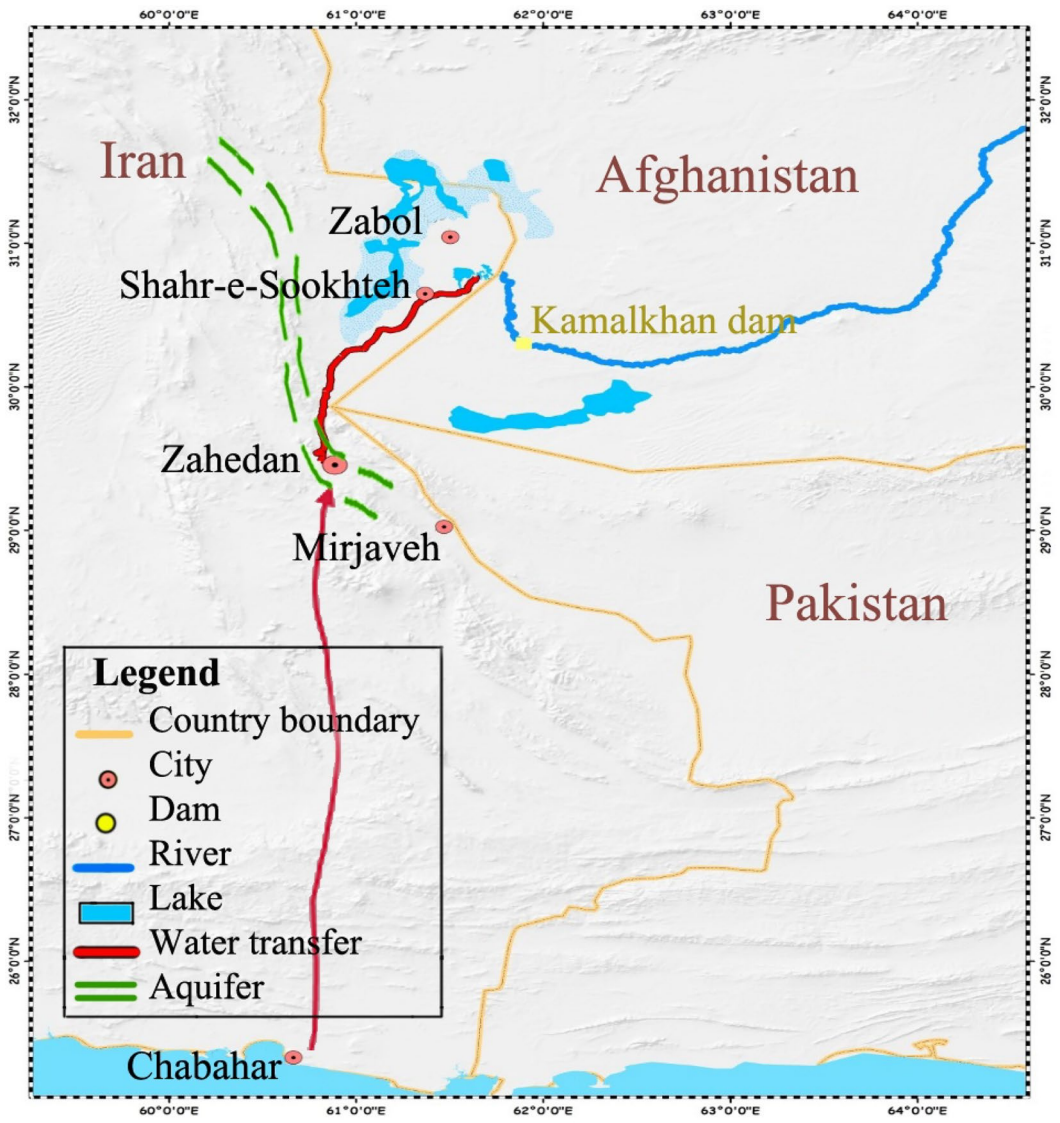
Location of water resources in the study area (This figure was created in the Quantum GIS, version 3.12.2).

Sistan Region’s water resources consist of the Helmand River, Gezik-Harmak aquifer, and desalinated sea water from Oman Sea (Fig. 3). Although annual flow of Helmand River is 7500 mcm, Iran's share in a normal year is determined as 820 mcm^35^, which reduces proportionally in dry years. Long-term series of annual Helmand inflow to Sistan shows that an average of 722 mcm has been received by Iran. Helmand River discharges into Hamoun Wetland which is being dried out in recent years because of prolonged dry periods and several water diversion projects in Afghanistan and Iran. Because of considerable decrease in Helmand inflows, a number of substituting water resources have been defined in Iran. One of them which is being studied is transferring 50 mcm water from the Oman Sea to the Sistan Region per year^34^. Gezik-Harmak aquifer is another source of water that has not been exploited yet^34^. The location of the aquifer is illustrated in Fig. 3. The exploitable water from the aquifer is estimated to be 15 mcm per year.

#### Determination of EAW resources

With the population equal to 394,029 at the target year (our vision in 2035), the water need for drinking and sanitation is estimated as 42 mcm per year. Although the yearly environmental needs of the Hamoun Wetlands in the Iranian side are estimated to be more than 5000 mcm^36^, a minimum amount of 181 mcm has been required to protect part of dust prone areas^37^. This minimum value has been determined by distinguishing high risk dusty areas that contribute to more than 80% dust production. Based on the climatological data, about 30% of water collected in the Sistan reservoirs are lost through evaporation. It means that evaporation consists of 218 mcm of Sistan’s water resources^38^. The rest of water resources is considered as an economic commodity, so it can be reallocated to the economic sectors to improve Sistan’s GDP compared to the country’s average value over a certain period of time (see "Optimal water allocation to job classes" Section). Table 1 reports water resources and the calculated EAW of Sistan.

**Table 1:** Water resources and water use in Sistan Region (mcm).

Water resources			Non-economic water use			Economic water use
Helmand River	Aquifer	See water desalination	Potable	Environment (dust control)	Evaporation	EAW
722	15	50	42	181	218	346

#### Resources required for job creation

About 20% of water resources of Sistan Region is presently consumed in service units such as hotels, restaurants, supermarkets, educational and health institutions, etc.^34^. Knowing the amount of water consumption in each of the main economic sectors of Sistan (agriculture, industry, and services) and the number of employees in each sector, one can determine the amount of water required for creating one job in every sector (Table 2). Similarly, having the GDP reported for every sector and water consumption values, the intensity of water consumption to produce one billion Rial (B-Ri) GDP (US Dollar (USD) to Iran Rial (Ri) exchange rate in 2016 is 30,144.68 Ri) can be calculated for every sector (Table 2). The table also includes the required capital for creation of one job that are simply calculated using the total investment required in every sector and the employment data and statistics extracted from Iran’s Statistics Center^30,39,40^, Iran’s Management and Planning Organization^34^, and Iran’s Agriculture Ministry^41^.

**Table 2:** Water productivity and required resources for creation of one job in Iran and SB in the economic sectors.

Economic sector	Region	Intensity of water consumption (m^3^/B-Ri)	Per job water needed (m^3^ per capita)	Per job capital needed (B-Ri)
Agriculture	Country	54,300	20,978	1.4
	SB	63,397	25,860	
	Ratio of SB to country	1.17	1.23	
Service	Country	172	121	1.05
	SB	293	151	
	Ratio of SB to country	1.7	1.26	
Industry	Country	1006	347	2.9
	SB	1445	500	
	Ratio of SB to country	1.44	1.44	

### Optimal water allocation to job classes

#### Input data estimation

Job classes are defined according to the extracted programs from spatial planning studies which are based on the potentials of the Sistan Region and the current activities in the region. Accordingly, job classes in the agricultural sector include 7 classes: (1) wheat, (2) barley, (3) oilseeds, (4) vegetables (cucumber, tomato, and onion), (5) forage (alfalfa), (6) melons, and (7) grapes. The first two classes are strategic crops that should be included in any development plan. A minimum production level of class 5 is necessary for the animals use, and classes 3, 4, 6, and 7 have been directly discussed in spatial planning studies of Sistan for more investment (see "Determining the development potentials and programs" Section). It should be noted that in the current situation, other crops in Sistan Region covers less than 1% of the area under cultivation, so they have been neglected in this study^39^.

On the other hand, according to the spatial planning studies (see "Determining the development potentials and programs" Section) the industrial sector includes 7 job classes: (1) food and beverage, (2) textile, (3) cellulose, (4) chemical, (5) non-metallic mineral, (6) metal, and (7) machinery tools.

According to these studies "Determining the development potentials and programs" Section, in the service sector, investing on the tourism and recreational activities has been emphasized. Overall, agricultural and industrial development necessitates job creation in many parts of the service sector of a society such as police, banking and tax affairs, sanitation, education, etc. Because of high variety of job classes in the service sector and lack of detailed information on different parts, service sector is considered herein as a lumped single class.

Based on the spatial planning studies of SB^34^ and available data and statistics of Agriculture Ministry^41^, area under cultivation, number of employees, crop yield, net irrigation demand, and GDP values of Sistan Region were extracted and presented in Table 3.

**Table 3:** Details of job classes of the agricultural sector in Sistan Region (2020).

	Job class	Area (ha)	Job no	Crop yield (ton/ha)	Net water demand (m^3^/ha)	GDP (B-Ri)	Annual growth rate (%)	Relative advantage coefficient
1	Wheat	37,214	5208	1.82	3520	932.86	0.71	0.70
2	Barely	15,602	1775	1.69	2920	295.32	2.95	0.60
3	Oilseeds	35.81	6	0.6	5240	0.548	5.93	0.50
4	Vegetables	6010	11,577	51	17,060	2375.45	6.30	1.00
5	Alfalfa	2270	1926	19.2	23,200	343.05	1.24	0.65
6	Melons	4373	1903	17.5	15,640	570.13	6.30	0.80
7	Grapes	1111	2051	8.1	22,110	682.81	26.06	1.50
Total/Average		66,616	24,447			5200.17	7.07	0.82

According to Table 3, total area under cultivation is 66,616 ha, GDP of agricultural sector is 5200 B-Ri, and the number of employees is 24,447 (about 29% of the Sistan’s employees). Under this cropping pattern, the total water use in the agriculture sector is estimated as 634 mcm. The annual growth rate of each job class from 2014 to 2020 in SB has been calculated and presented in Table 3. The relative advantage coefficients are also extracted from the spatial planning studies of SB^34^ as listed in the last column of the table. In addition, to be able to consider deficit irrigation in the agricultural sector, the crop yield response factor to water deficit (*K*_*y*_) was extracted^42^ and employed in the optimization model (Table 4).

**Table 4:** Response coefficient of crop yield in deficit irrigation^42^.

Crop	Wheat	Barely	Oilseed	Vegetables	Alfalfa	Weeds	Grapes
*K*_*y*_	1.05	1.1	0.95	1.1	1.1	1.1	1.5

Similarly, the characteristics of the mentioned 7 industrial job classes (Table 5) were extracted from different references^30,34,40^. A total of 38 large industrial units are active in the Sistan Region. Likewise, most of employees are in the industry sector. The largest number of industries is related to the chemical and food industries. However, the employment potential of the textile industry and its water consumption are relatively high. Among all the industrial job classes, the metal class has the highest GDP. According to Table 5, it is estimated that about 2.1 mcm water is consumed in this sector, which is very low compared to the agricultural water consumption (0.5% of Sistan water resources) indicating insufficient attention to industrial development in Sistan Region. With a total of 6305 industrial jobs, the share of employment is about 8%. The GDP value for this sector is 2465 B-Ri, which is about half of that for the agricultural sector. This shows the importance and effective role of industrial development in Sistan Region. The annual growth rate and relative advantage coefficient of each job class have also been calculated and presented in Table 5^34^.

**Table 5:** Details of 7 job classes of the industry sector in Sistan Region.

	Job class	no. of industrial units	no. of jobs	Job (Person/Unit)	Water use (m^3^/capita)	GDP B-Ri	Annual growth rate (%)	Relative advantage coefficient
1	Food and beverage	11	2155	196	290.41	641.14	1.92	1.22
2	Textile	3	1066	355	208.78	437.81	0.70	0.47
3	Cellulose	2	210	105	215.41	49.6	1.01	0.66
4	Chemical	16	2260	141	324.88	353.42	1.57	0.09
5	Non-metallic mineral	1	114	114	649.76	139.02	2.03	1.37
6	Metal	4	456	114	957.11	806.52	3.72	0.32
7	Machinery tools	1	46	46	235.54	37.96	1.31	0.06
Sum/Average		38	6305	153	411.69	2,465.43	1.75	0.6*

#### Constraints and objective function

Having calculated the redistributed water resources to the industrial and agricultural sectors, the problem of optimal allocation of water to different job classes is formulated in the form of an optimization problem. Suppose *m* is the number of activities (job classes) in three sectors of industry, service, and agriculture selected based on the spatial planning studies, *n*_*i*_ (*i* = 1,2,…, *m*) is the number of people employed in each of the activities, *UW*_*i*_ is the water requirement of activity *i* per job, *UCap*_*i*_ is the capital required to create one job of class *i* (Tables 3 and 4), *UGDP*_*i*_ is the share of total GDP per job for job class *i*﻿, and *Pr*_*i*_ is the production amount per unit of water consumption (water productivity) for job class *i*.

The problem is defined so as to determine optimal allocation of ﻿total number of jobs, $T\_Job$, to *m* activities that have been defined in different classes. Percentage of the occupied population ($r_{p}$) is equal to:

1 $$r_{p} { = }\frac{T\_Job}{{{\mathbf{{\mathbb{P}} }}}} \times 100$$

where ${\mathbf{\mathbb{P}}}$ is total population of the study area at the target year, and *T_Job* = $\sum\nolimits_{i = 1}^{m} {n_{i} }$ is the total number of jobs created in all job classes, which is less than the total population ready to work (*P*) if immigration is neglected.

2 $$T\_Job \le P$$

Likewise, the total required capital (*T_Capital*) and the total GDP (*T_GDP*) as a result of job allocation can be calculated as follows:

3 $$T\_Capital{ = }\sum\limits_{i = 1}^{m} {Cap_{i} } = \sum\limits_{i = 1}^{m} {n_{i} .UCap_{i} }$$

4 $$T\_GDP{ = }\sum\limits_{i = 1}^{m} {GDP_{i} } = \sum\limits_{i = 1}^{m} {n_{i} .UGDP_{i} }$$

where *Cap*_*i*_ is the required capital for creation of job class *i* (see Table 2) and *GDP*_*i*_ is the produced *GDP* in job class *i* (see Tables 3 and 4). As one of the goals of optimizing water allocations, the model will search for a solution where Sistan’s average UGDP ($\overline{UGDP}$_sis_) exceeds Iran’s average UGDP ($\overline{UGDP}$_ave_). This constraint guarantees that a minimum acceptable level of development in the less-developed Sistan Region, ﻿compared to that of the average of the country, is reached until the target year.

5 $$\overline{UGDP}_{sis} \ge \overline{UGDP}_{ave}$$

Moreover, the total production (*T_Prod*) can be determined as written below:

6 $${\text{T\_Prod = }}\sum\limits_{i = 1}^{m} {{\text{Prod}}_{i} } { = }\sum\limits_{i = 1}^{m} {n_{i} UW_{i} {\text{Pr}}_{i} \alpha_{i} }$$

where *Pr*_*i*_ is the production as a result of one unit of water used in class *i*, and *α* is the coefficient of crop yield reduction in agricultural sector due to deficit irrigation.

In order to obtain the maximum benefit in different sectors from limited water resources, deficit irrigation is applied in agriculture. In deficit irrigation strategy crop yield reduces, so does the crop production. Here, the FAO method is used to estimate the crop yield resulting from deficit irrigation as follows^42^:

7 $$\alpha = \frac{{Y_{a} }}{{Y_{p} }}{ = 1} - K_{y} \left( {1 - \frac{{ET_{a} }}{{ET_{p} }}} \right)$$

where *Y*_*a*_ and *Y*_*p*_ are the actual and maximum crop yield, respectively, and *ET*_*p*_ and *ET*_*a*_ are respectively the potential and actual evapotranspiration. Note that for the industrial and services job classes, *α* is considered as 1.0. It is worth mentioning that the total water use must be less than EAW (*V*_*p*_). On the other hand, if the objective function is minimization of the required financial investment, then the optimization model is forced to use a percentage ($\xi$) of EAW ($\xi .{\text{V}}_{p}$) as presented below:

8 $$\xi .{\text{V}}_{p} \le \sum\limits_{i = 1}^{m} {\left( {1 - \eta_{j} } \right)n_{i} DW_{i} } \le {\text{V}}_{p} \,\,\,\,\,\,\,\,\,\forall j = 1..3$$

where* j* denotes the three economic sectors, and $\eta_{j}$ is the target water consumption improvement coefficient in sector *j* specified according to the performed spatial planning studies where an improvement in the irrigation efficiency is expected. In this study, a 10% improvement is considered, although it is not a certain parameter. This constraint can provide different development plans associated with different levels of investment. This study has considered $\xi$ = 0.8 which means an optimal development plan corresponding to the use of at least 80% of EAW is being seeked. According to the spatial planning documents^34^, the maximum area that can potentially be cultivated (*M_Area*) in Sistan region is 46,000 ha; therefore: 

9 $$\sum\limits_{i = 1}^{{m_{1} }} {Area_{i} } \le M\_Area$$

where *m*_*1*_ is the number of agricultural classes (m_1_ = 7) and *Area*_*i*_ is the area under cultivation for crop *i*﻿. In addition, in terms of social acceptability, the annual change in every job class (*β*_*i*_) should not be higher than the maximum growth rate experienced in the past (*M_β*):

10 $$\beta_{i} \le M\_\beta$$

Also, there is a set of strategic products (*A*_1_) in the region for which a minimum level of production of which should be guaranteed:

11 $${\text{Prod}}\left( {A_{1} } \right) \ge \zeta .D_{Str}$$

where $\zeta$ is the coefficient of self-sufficiency for strategic products (here 50%) and *D*_*str*_ is the total demand for these products (wheat and barley) in the region. With annual demand of 230 kg/capita of strategic products (170 kg wheat and 50 kg barely)^34^, *D*_*str*_ will be about 90,600 ton in the target year. It is worth noting that fishery and livestock activities are omitted due to their small shares in the employment condition of the region. However, these activities were part of Sistan economy before 1997 when Hamoun wetland was in its normal condition^43^.

Additionally, the relative comparative advantage of job class *i* (*Be*_*i*_) is considered in the following objective functions of the model:

12 $$Max:Job{ = }\sum\limits_{i = 1}^{m} {Be_{i} \times n_{i} }$$

13 $$Max:GDP{ = }\sum\limits_{i = 1}^{m} {Be_{i} n_{i} UGDP_{i} }$$

14 $$Min:Capital{ = }\sum\limits_{i = 1}^{m} {Be_{i} n_{i} UCap_{i} }$$

The optimization model introduced allocates the fixed amount of EAW and other natural resources to *T_Job* with the aim of maximizing employment and GDP or minimizing the required financial investment. The key decision variables of the model (*n*_*i*_) are integer variables, so the model formulation is a mixed integer nonlinear program which is non-convex due to yield functions. Consequently, it is not easy to solve the model using classical gradient-based optimization algorithms. Therefore, the genetic algorithm (GA) is used to solve the optimization model presented above. The model consists of 15 main decision variables and the GA searches for the optimal solution using single-point crossover, roulette wheels selection, and mutation operators. Infeasible solutions are penalized by considering penalty terms regarding the unsatisfied constraints in the objective function.

## Results

Equation (5) guarantees that the Sistan’s GDP will approach to that of the country’s mean value due to the WCGD-based model. Tables 3 and 5 show the values of GDP in the SB in the main sectors. It is seen that in the agricultural sector, the per capita GDP values for SB and the country are close to each other, but there is a large gap between these values for the industry sector. In the presented model, EAW is the pillar for development plan. According to "Analysis of water resources and water demand" Section and Table 1, there are three major sources of water in Sistan: Helmand River, water desalination, and Gezik-Harmak aquifer. As the two last sources of water have not yet been used, three scenarios of available EAW resources are defined as follows:

- S1: Supplying the economic sectors only by Helmand River water resources (EAW = 281 mcm);
- S2: Supplying the economic sectors by Helmand River and the aquifer water resoutces (EAW = 296 mcm);
- S3: Supplying the economic sectors by Helmand River, aquifer, and desalination water resources (EAW = 346 mcm).

Accordingly, for every scenario, the optimal distribution of water in different job axes is determined. Use of total amount of available water has been guaranteed in the optimal model by Eq. (8). With these amounts of EAW, the GA-based optimization models are solved and the results are presented.

**Figure 4:**
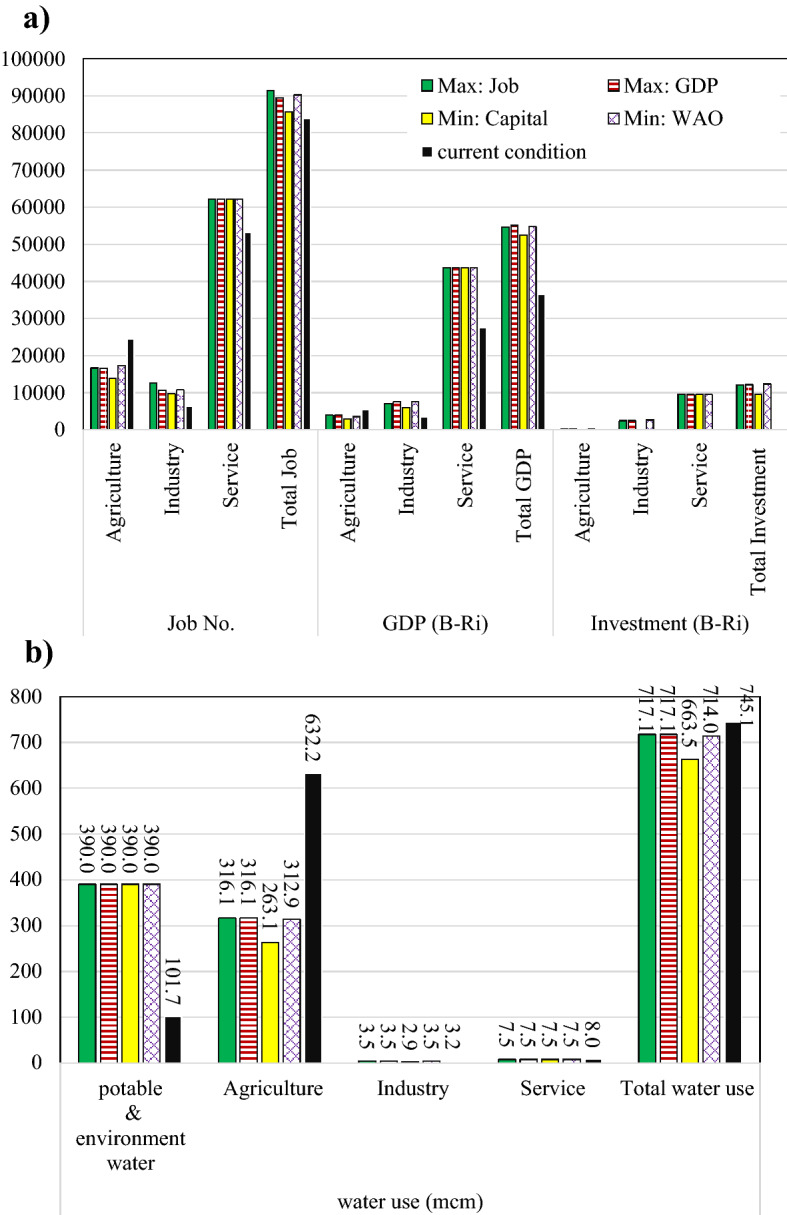
Optimization model results of S3 for (**a**) number of jobs, GDP, the required investment, and (**b**) water use.

As mentioned previously, the service sector is considered as a lumped sector having a single job class in the optimization model. The optimization model is solved separately for each of three mentioned objective functions (Eqs. 12–14) in addition to a model combining the objective funtions by a weighted average﻿ (WA) objective function. The results including job creation, GDP production, and required capital for development corresponding to the third scenario (S3) are presented in Fig. 4. It can be seen that, the optimal number of jobs and GDP value increase to 91,447 persons and 55,119 B-Ri, respectively, while meeting potable and environmental water demands is guaranteed. Number of jobs in the agricultural sector has decreased because of reducing water use in this sector. Accordingly, more jobs have been created in industry and service sectors. Out of total jobs, 68, 13, and 19% are devoted to service, industrial, and agricultural sectors, respectively. The total jobs created in the region has grown up by 8.9% and GDP has increased by 51.1% where 79, 14, and 7% of GDP correspond to service, industry, and agriculture sectors, respectively.

Similarly, the lowest possible amount of investment while utilizing at least 80% of available water resources is equal to 9558 B-Ri, of which more than 99% is for the service sector and less than 1% is for the industry sector. According to the results, no additional investment is spent on the agricultural sector as further development in this sector should be avoided where a decrease in the level of cultivation area is proposed for all the crops.

**Figure 5:**
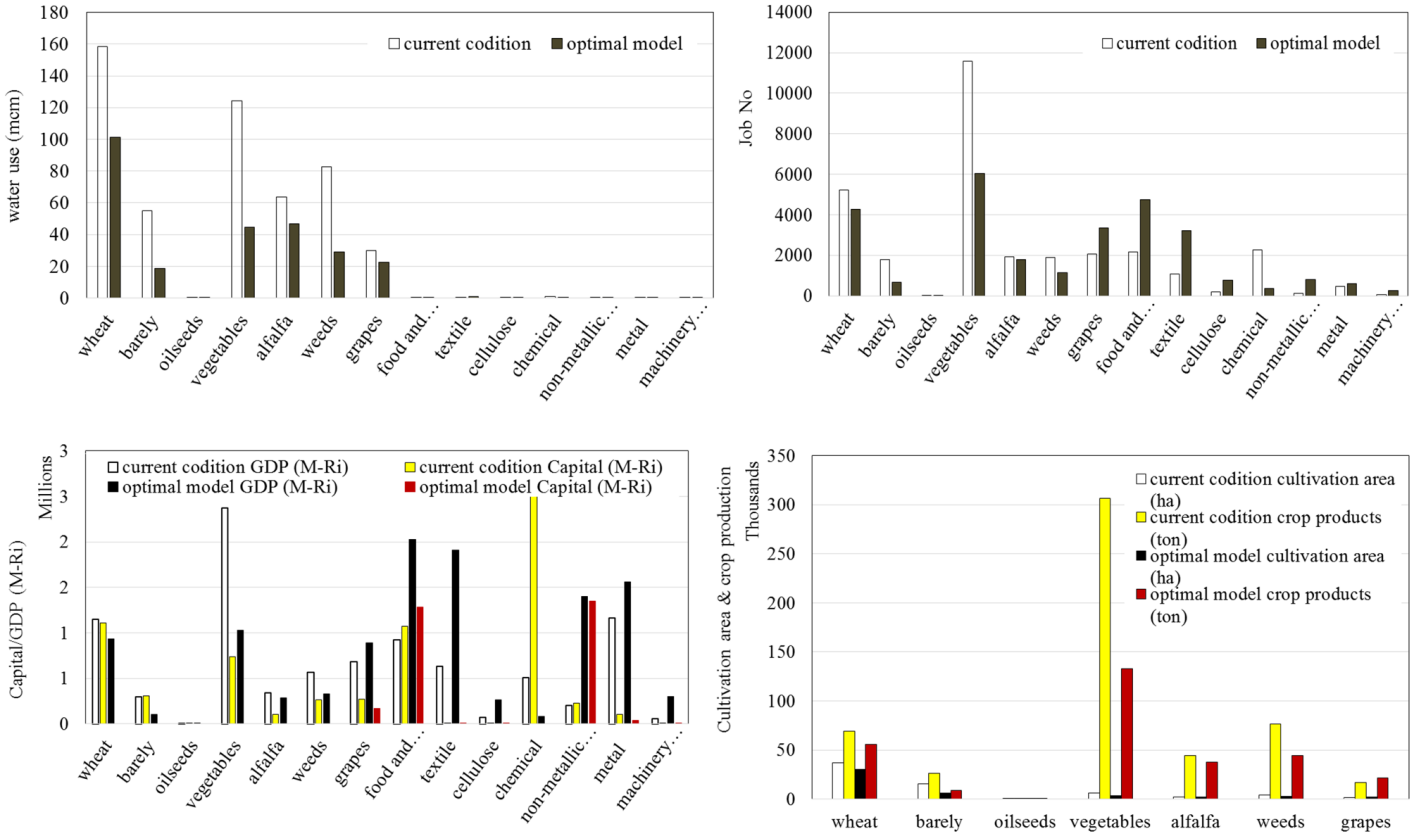
Comparison of current condition with that proposed by WCGD framework under scenario S3 of EAW and the WA objective function.

Figure 5 presents more detailed results of the optimization model for S3 corresponding to the fourth objective function (WAO) where the values of water use, job number, GDP, and the required capital are illustrated for 14 agriculture and industry job classes compared to those of the current condition.

**Figure 6:**
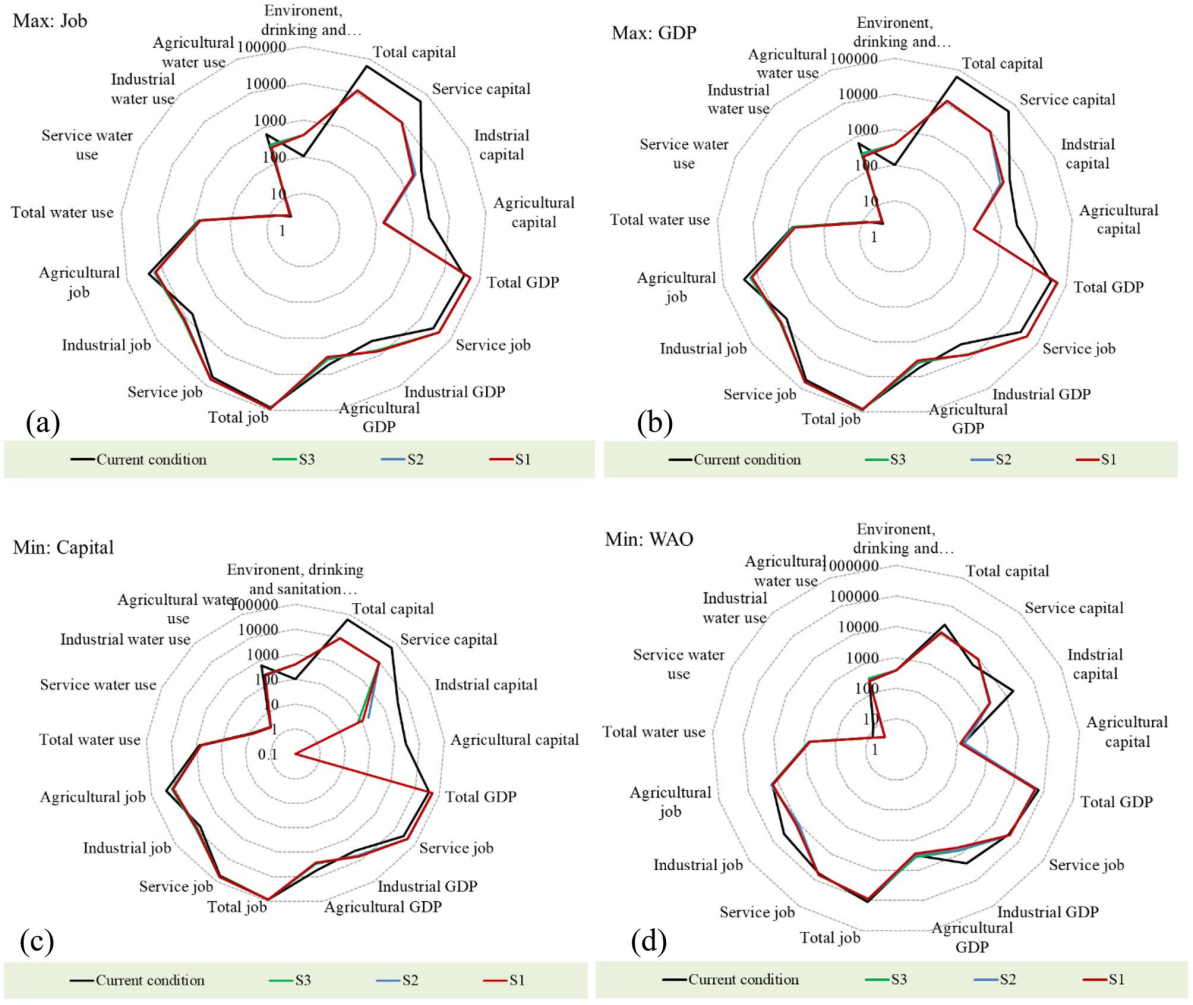
Amount of water use, number of jobs, GDP production, and the capital required under different scenarios for different objective functions: (**a**) Max: Job, (**b**) Max: GDP, (**c**) Min: Capital, and (**d**) Min: WA function.

Figure 6 shows the results of the optimization model obtained for four different objective functions compared to the existing conditions. In all the defined scenarios of EAW, potable and environmental water demands are fully met. Figure 6a shows that the maximum created job number is 91,447 corresponding to scenario S3. In this situation, despite a 3.7% reduction in water use and meeting the total demand of the environment, available jobs in the region increase by 8.9%. According to Fig. 6b, due to the development of industry and service sectors, GDP increases significantly with an investment of 12,148 B-Ri (+ 51% growth), which is in line with the poverty alleviation strategy followed by the region’s spatial planning goals. In addition, Fig. 6c shows that with a minimum capital of 5558 B-Ri (63 B-Ri in industry and 9495 B-Ri in service), employment and GDP improve more than 2.2% and 44%, respectively. Under this scenario, 10.9% reduction of water use in the economic sectors is realized without compromizing the vital DSE water needs.

According to Fig. 6, for all scenarios, the optimal investment on the agricultural sector is zero when the objective function is to minimize the required investment. However, for other objective functions, due to the change in the cultivation pattern, some investment is required. The WCGD-based approach replaces the present crops with those having higher yields and are more profitable, while accounting for the constraints imposed on the maximum level of cropping pattern change.

**Figure 7:**
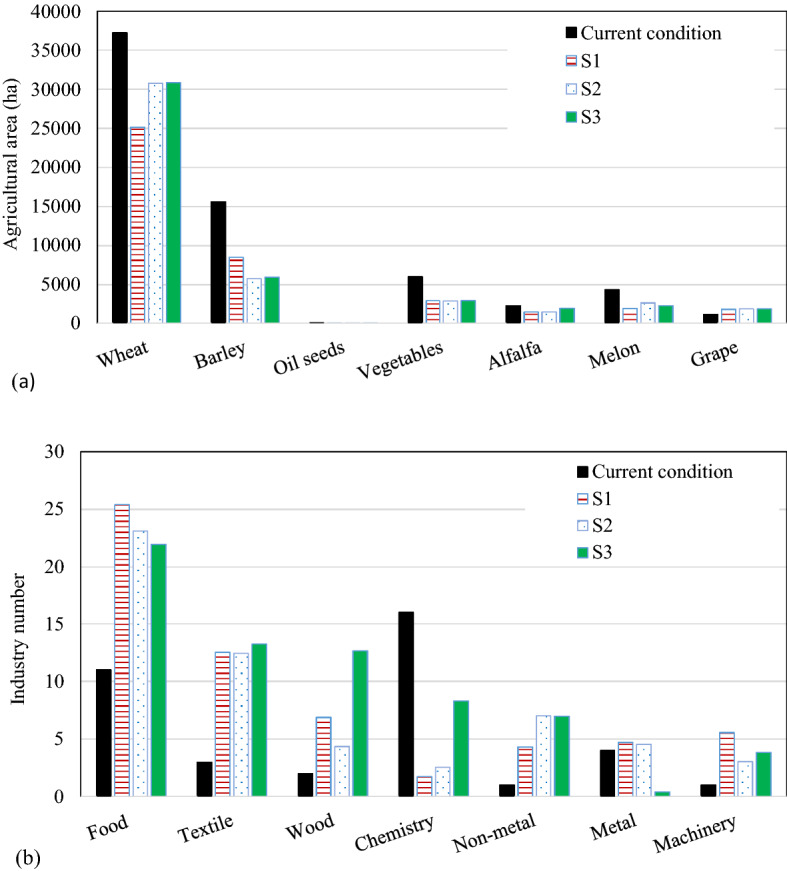
(**a**) Comparison of the change in the cultivation area in the agricultural sector and (**b**) change in the number of industrial units under different development scenarios of EAW and the existing conditions.

Figure 7a shows the optimal cultivation pattern in different scenarios while the objective function is Max: Job. It is seen that the area under cultivation for all crops except grapes has decreased. The reasons are the potential for the extention of grape production in the region according to the region’s spatial planning strategies and the fact that it is more profitable than other agricultural products. In addition, the production of strategic crops of wheat and barley together has been maintained up to 50% of their current needs. For scenarios S2 and S3 compared to S1, the area under cultivation of wheet has decreased less than that of barely.

Figure 7b shows the changes realized in the industrial sector. More changes are observed compared to the agricultural sector. For S3, more water has been devoted to chemistry and wood industries. Although the proportion of changes in job classes is almost the same, the chemical industry is replaced in all the scenarios by food, textile, and wood industries, because of more job creation per one unit of water use in these latter industries. Generally speaking, the food industry has been more attractive than other industries in terms of job creation.

## Discussion

In this research, considering three scenarios of economically-allocable water (EAW) availability in the Sistan Region, the total number of jobs, GDP, and the required capital for development were determined toward meeting sustainable development goals in the low-developed Sistan Region, Iran. The EAW concept guarantees water supply for drinkiung, sanitory, and environment (DSE) as a necessity  after which the rest of water resources could be used as an economic goods for producing GDP in three economic sectors of agriculture, industry, and service. As the simplistic assumptions made, a constant share has been considered for DSE sectors, and the uncertainty in water resources availability has not been accounted for in this study, so the minimum inflow from Helmand River to Sustan region has been used in the proposed water-constrained green developemnt (WCGD) framework to increase the applicability of the solutions obtained. Under the mentioned assumption and considering different sources of avilable water that can possibly utilized, three possible EAW values were used as the main input to the developed optimization model to find optimal shares of water and other resources allocated to 15 job classes of the economic sectors while neglecting some other job classes because of their limited shares in GDP of the region. Also, the service sector was considered as a whole with a single job class, because it involves several job classes with almost the same consumption of water and other related natural resources. Based upon the optimization model results obtained, despite the reduction of water consumption and full provision of the DSE’s share, employment in the region increases by about 10% of which respectively 68 and 14% of employees work for service and industry sectors and the remaining 18% work for the agriculture sector. Additionally, the GDP increases from 36.482 B-Ri to 55,119 B-Ri, which the shares of 79, 14, and 7% for the service, industry and agriculture sectors, respectively. The results have been obtained under several strict limitations and constraints on the upper or lower bounds of some parameters such the growth rate of job classes, magnitude of possible change in current irrigation practices, production level of strategic crops, and available water, land, and human resources. The main concern is the issue of food security in the region as the total agricultural cultivated area decreases under the proposed development scenarios. However, this concern may be resolved by importing the required food. Overall, the results of the optimization model show that by changing the development trend according to the WCGD framework and modifying the existing activities, more socio-economically ﻿effective water allocation policies can be achieved without destructive environmental effects. Undoubtedly, increased GDP and job availability resulted are positive socio-economic indicators in the Sistan Region. It is worth mentioning that the results obtained are based on the assumptions made for a number of uncertain parameters including: ﻿“growth rate of job classes”, “self-sufficiency coefficient for the strategic products”, and “water consumption improvement coefficient”﻿. The first parameter value was considered to be less than the maximum growth rate experienced in the past. For the agriculture, industry, and service sectors, the growth rates were constrained to 6.5, 6.5, and 61% annually. Also, the coefficient of self-sufficiency for wheat and barley were considered as 50% of their normal demand in the region. Finally, the water consumption improvement coefficient was supposed to be 10% in the agriculture sector. All these parameter values are uncertain, so different results will be obtained if they change.

## Conclusions

Sistan is highly dependent on the Helmand River inflows in terms of water resources. The 1973 Water Treaty defines how to share water between Afghanistan and Iran, and the limited available water should be well distributed among consumers in both countries. In the past years, the inflow to the Hamoun wetland has sharply decreased due to prolonged droughts, upstream development plans in Afghanestan, and inefficient water use in Afghanistan and Iran. Unfortunately, per capita GDP in Sistan is one third of the Iran’s average, and the population growth rate in Sistan is negative. This situation makes the need for revising the development pattern in the region more apparent. In this research, a solution to the problem of reforming the development pattern of the Sistan Region was introduced by proposing the new water-constrained green development (WCGD) framework that pays special attention to the required water for drinking, sanitation, and environment (DSE), as the human rights. This framework carefully considers the development potentials in accordance with spatial planning studies and finds the optimal water allocation pattern to economic sectors under constraints of available economically-allocable water (EAW) and other resources. EAW is the total water resources in excess of the required water for DSE that can contributes to the development process. The WCGD-based inter-sectoral water allocation problem ﻿in the low-developed Sistan region was formulated as a mixed integer nonlinear program and was solved by genetic algorithms to optimally allocate water and land resources to 15 job classes in the sectors of agriculture, industry, and service. According to the results, optimal reallocation of EAW resources can improve the region’s employment condition without any negative impact on the environment and society. Also, under the proposed revised water allocation policies, Sistan’s GDP index can reach the country’s average GDP index. There will be however some challenges in this process. For example, it is not easy to change people’s life style and their socio-economic activities towards what proposed by the WCGD framework in a short period of time, so implementation of the framework calls for capacity building and additional social, cultural, legal, and institutional provisions.

## Data Availability

The datasets generated during and/or analyzed during the current study are available from the corresponding author on reasonable request.

## References

[1] Goswami A (2016). Economic Modeling, Analysis, and Policy for Sustainability.

[2] WCED (World Commission on Environmental Development). Our common future [Brundtland report]. UN, New York (1987).

[3] World Bank. Toward a Green, Clean, and Resilient World for All, A World Bank Group Environment Strategy 2012–2022 (2012). www.worldbank.org/environment

[4] UNEP. Towards a Green Economy: Pathways to Sustainable Development and Poverty Eradication (2011). www.unep.org/greeneconomy.

[5] Loucks DP (2017). Managing water as a critical componnets of a changing world. Water Resour. Manag..

[6] Stanton MCB, Roelich K (2021). Decision making under deep uncertainties: A review of the applicability of methods in practice. Technol. Forecast. Soc. Change.

[7] Adams VM (2017). Making time for space: The critical role of spatial planning in adapting natural resource management to climate change. Environ. Sci. Policy.

[8] Meerow S, Newell JP (2017). Spatial planning for multifunctional green infrastructure: Growing resilience in Detroit. Landsc. Urban Plan..

[9] Novak M (2022). The role of spatial plans adopted at the local level in the spatial planning systems of central and eastern European countries. Water.

[10] Carter J (2007). Spatial planning, water and the Water Framework Directive: Insights from theory and practice. Geogr. J..

[11] Hargreaves AJ, Farmani R, Ward S, Butler D (2019). Modelling the future impacts of urban spatial planning on the viability of alternative water supply. Water Res..

[12] Gallagher L (2016). Embracing risk, uncertainty and water allocation reform when planning for green growth. Aquat. Procedia.

[13] Hu Z, Wei C, Yao L (2016). A multi-objective optimization model with conditional value-at-risk. J. Hydrol..

[14] Wang J (2016). Optimal allocation of water resources based on water supply security. Water.

[15] Azadi S, Nozari H, Ghanbarian B, Marofi S (2022). Optimizing cropping pattern to improve the performance of irrigation network using system dynamics-Powell algorithm. Environ. Sci. Pollut. Res..

[16] Barati K, Abedi Koupai J, Darvishi E, Azari A, Yousefi A (2020). Cropping pattern optimization using system dynamics approach and multi-objective mathematical programming. J. Agric. Sci. Technol..

[17] Osama S, Elkholy M, Kansoh RK (2017). Optimization of the cropping pattern in Egypt. Alex. Eng. J..

[18] Hao LN, Su XL, Singh VP (2018). Cropping pattern optimization considering uncertainty of water availability and water saving potential. Int. J. Agric. Biol. Eng..

[19] Yazdandoost F, Razavi H, Izadi A (2022). Optimization of agricultural patterns based on virtual water considerations through integrated water resources management modeling. Int. J. River Basin Manag..

[20] Jin Z, Ge D (2021). Optimization of sustainable land use management in water source area using water quality dynamic monitoring model. Comput. Intell. Neurosci..

[21] Ajudiya B, Yadav SM, Majumdar PK (2022). Optimization of cropping patterns in the command area of multiple reservoir system using TLBO algorithm. ISH J. Hydraul. Eng..

[22] Habibi Davijani M, Banihabib ME, Nadja A (2016). Optimization model for the allocation of water resources based on the maximization of employment in the agriculture and industry sectors. J. Hydrol..

[23] Amini Fasakhodi A, Nouri SH, Amini M (2010). Water resources sustainability and optimal cropping pattern in farming systems; a multi-objective fractional goal programming approach. Water Resour. Manag..

[24] Viola I, Pontrandolfi A, Manelli A (2016). The employment crisis and green orientation in agriculture: New educational models. Agric. Agric. Sci. Procedia.

[25] Cecere G, Mazzanti M (2017). Green jobs and eco-innovations in European SMEs. Resour. Energy Econ..

[26] He L, Wang S, Peng C, Tan Q (2018). Optimization of water consumption distribution based on crop suitability in the middle reaches of Heihe River. Sustainability.

[27] Farrokhzadeh S (2022). sustainable water resources management in an arid area using a coupled optimization-simulation modeling. Water.

[28] WAPCOS (Water and Power Development Consultancy Services). Lower Helmand valley development, Water and Power Development Consultancy Services (India) Ltd. (1975).

[29] UNDP (United Nations Development Programme). Enhancing integrated natural resource management for the restoration of wetland ecosystems and support to alternative livelihoods development of local communities. Project Communications, Partnership and Knowledge Management Expert (2022).

[30] Iran’s Statistics Center. National Population Census (2016). available at: https://www.amar.org.ir/

[31] Foltz RC (2016). Iran in World History.

[32] Rouhi-Moghaddam E, Sargazy E, Gholamalizadeh A (2015). Ecological properties of Tamarix habitats in Sistan plain, Iran. Ecopersia.

[33] Mir R, Azizyan G, Massah A, Gohari A (2022). Fossil water: Last resort to resolve long-standing water scarcity?. Agric. Water Manag..

[34] Iran’s Management and Planning Organization. Spatial planning of Sistan-Baluchestan province, Supreme Council of Spatial Planning, Office of the President, Tehran, Iran (2020).

[35] Helmand Water Treaty. The Afghan-Iranian Helmand River-Water Treaty of 1973 (1973). https://www.internationalwaterlaw.org/documents/regionaldocs/1973_Helmand_River_Water_Treaty-Afghanistan-Iran.pdf

[36] Iran’s Ministry of Energy. Invulnerable Sistan: Strengthening of Siatan Plain, A report by the Ministry of Energy, Tehran, Iran (2020).

[37] Iran’s Department of Environment. National document of dust. Department of Environment, Office of the President, Tehran, Iran (2016).

[38] WRI (Water Research Institute). Integrated Water Resources Management for the Sistan Closed Inland Delta, Iran. Main Report (2006).

[39] Iran’s Statistics Center. National Agriculture Census (2014). Available at: https://www.amar.org.ir/

[40] Iran’s Statistics Center. Statistical yearbook of the country (2020). Available at: https://www.amar.org.ir/

[41] Iran’s Agriculture Ministry. Annual Agricultural Statistics (2014–2020). Available at https://www.maj.ir/page-amar/FA/65/form/pId3352

[42] FAO. Deficit Irrigation Practices. Water Reports No. 22. Rome (2002).

[43] Weier, J. From wetlnd to waste land: the distruction of the Hamoun Oasis, Earth observatory. Nasa (2002).

